# Homogeneous nucleation and microstructure evolution in million-atom molecular dynamics simulation

**DOI:** 10.1038/srep13534

**Published:** 2015-08-27

**Authors:** Yasushi Shibuta, Kanae Oguchi, Tomohiro Takaki, Munekazu Ohno

**Affiliations:** 1 Department of Materials Engineering, The University of Tokyo, 7-3-1 Hongo, Bunkyo-ku, Tokyo 113-8656, Japan; 2 Faculty of Mechanical Engineering, Kyoto Institute of Technology, Matsugasaki, Sakyo-ku, Kyoto 606-8585, Japan; 3 Division of Materials Science and Engineering, Faculty of Engineering, Hokkaido University, Kita 13 Nishi 8, Kita-ku, Sapporo, Hokkaido 060-8628, Japan

## Abstract

Homogeneous nucleation from an undercooled iron melt is investigated by the statistical sampling of million-atom molecular dynamics (MD) simulations performed on a graphics processing unit (GPU). Fifty independent instances of isothermal MD calculations with one million atoms in a quasi-two-dimensional cell over a nanosecond reveal that the nucleation rate and the incubation time of nucleation as functions of temperature have characteristic shapes with a nose at the critical temperature. This indicates that thermally activated homogeneous nucleation occurs spontaneously in MD simulations without any inducing factor, whereas most previous studies have employed factors such as pressure, surface effect, and continuous cooling to induce nucleation. Moreover, further calculations over ten nanoseconds capture the microstructure evolution on the order of tens of nanometers from the atomistic viewpoint and the grain growth exponent is directly estimated. Our novel approach based on the concept of “melting pots in a supercomputer” is opening a new phase in computational metallurgy with the aid of rapid advances in computational environments.

It is important to precisely control the microstructures of metals and alloys during solidification since they directly affect the properties of metal and alloy products. In spite of considerable effot from both fundamental and industrial viewpoints^1^,^2^, it is still not straightforward to control solidification microstructures since the solidification is affected by many aspects of physics such as heat transfer, convection, and solute diffusion. Moreover, it is difficult to observe these processes directly by an experimental approach. Therefore, computational studies have contributed to our understanding of the nature of the solidification process. Formerly, Monte Carlo (MC) simulations based on the Potts model^3^ were carried out to study the kinetics of grain growth^4^,^5^, and they became popular in the investigation of solidification, grain coarsening, and recrystallization^6^,^7^. In 1993, the phase-field model based on the variational principle of the Ginzburg-Landau free energy was successfully used to reproduce a dendrite structure^8^. Since then, the phase-field model^9^,^10^,^11^ has entered mainstream use for the simulation of solidification^12^, and its range of application now covers the competition between bundles of dendrites, including selection, regularity, and segregation^13^,^14^. In addition, molecular dynamics (MD) simulations have contributed to the estimation of thermodynamic properties related to solidification^15^,^16^,^17^ as well as to the understanding of morphological dynamics during solidification^18^,^19^. Computational metallurgy now covers a wide range of phenomena associated with solidification and microstructure evolution, assisted by the recent progress in high-performance computational environments.

One of the remaining problems in computational metallurgy is how to treat nucleation. In most previous phase-field and MC simulations on solidification and microstructure evolution, the nuclei in the melt are specified in advance as having a random distribution or are forcibly formed in line with an assumption based on classical nucleation theory. That is, it is not straightforward to investigate nucleation itself by performing phase-field and MC simulations. On the other hand, it is possible in principle to achieve nucleation in MD simulations if the undercooled melt is kept unchanged for a longer time than the incubation time of nucleation. Actually, the nucleation in small metal nanoparticles has been achieved in an MD simulation by continuous cooling^20^,^21^, although nucleation in the nanoparticles occurred only once or at most a few times. Moreover, there have been several pioneering works, in which the nucleation was achieved in a large-scale system with the aid of inducing factors such as pressure-induced^22^ and surface-induced solidification^23^. However, a broad spatiotemporal scale is required to achieve spontaneous (i.e., thermally activated) nucleation, which is computationally demanding. Therefore, it is not yet straightforward to investigate spontaneous homogeneous nucleation, even by MD simulation, without an inducing factor.

Under such circumstances, we have developed our own code for carrying out MD simulations on a graphics processing unit (GPU), which enables the handling of one million atoms in MD simulations over a period of nanoseconds and has a computation time of several days^24^,^25^. Using this code on a GPU architecture, we successfully revealed the spontaneous evolution of anisotropy in a solid nucleus during the solidification of iron comprising one million atoms^19^. These new insights obtained using the powerful MD tool inspired the idea of directly capturing the nature of nucleation by statistical sampling of a large-scale MD simulation. To this end, spontaneous nucleation from an undercooled melt of iron and the subsequent microstructure evolution are investigated by the statistical sampling of isothermal MD calculations with one million atoms performed on the GPU supercomputer, TSUBAME2.5.

## Results and Discussion

### Atomic configuration during nucleation and grain growth

**Figure 1:**
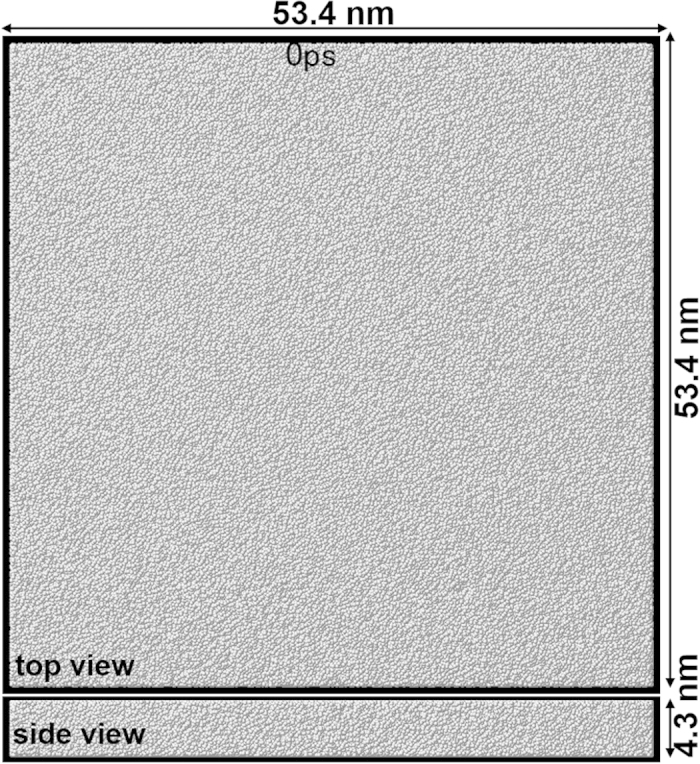
Initial configuration of the simulation cell filled with an iron melt. The system consists of 1,037,880 atoms in a cell of 53.4 × 4.3 × 53.4 nm with the periodic boundary condition.

Nucleation and subsequent grain growth are achieved by isothermal holding of a simulation cell filled with an iron melt consisting of 1,037,880 atoms (Fig. 1) at various undercooling temperatures between 900 and 1800 K with 100 K intervals. Since the melting point of bcc iron given by the Finnis−Sinclair (FS) potential employed here is 2400 K^26^, which is higher than the experimental value of 1811 K, temperatures are normalized by the melting point given by the FS potential, *T*_*m*_ = 2400 K.

**Figure 2:**
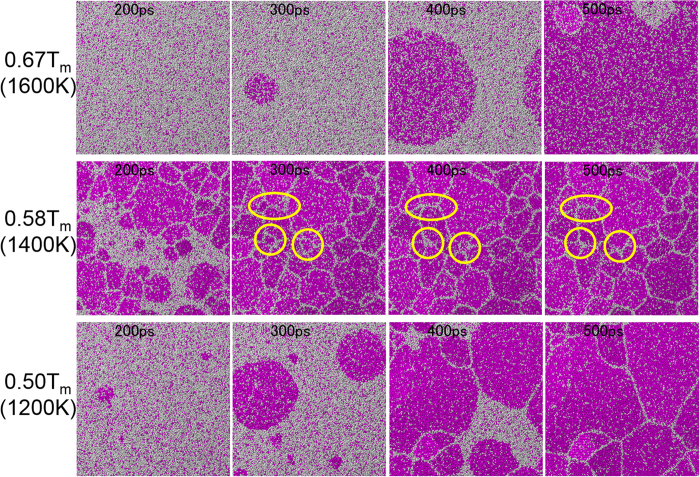
Snapshots of atomic configuration during nucleation, grain growth, and microstructure evolution at 0.67*T*_*m*_, 0.58 *T*_*m*_, and 0.50*T*_*m*_. Red and white spheres in this and other figures represent atoms with and without the bcc configuration, respectively. Yellow circles highlight small grains, which shrink and disappear as a result of grain coarsening.

Figure 2 shows snapshots of the atomic configuration during nucleation and subsequent grain growth at 0.67*T*_*m*_, 0.58*T*_*m*_, and 0.50*T*_*m*_, which are representative results from the five replicate calculations for each temperature. Videos of these processes are available in Supplementary Information. In the case of 0.67*T*_*m*_, one nucleus is nucleated at a time between 200 and 300 ps and grows into a spherical grain in the melt. Another nucleus is then formed near the edge of the spherical grain and is surrounded by the previously nucleated grain at 500 ps. On the other hand, many nuclei are simultaneously nucleated before 200 ps at 0.58*T*_*m*_, which results in the formation of a microstructure consisting of fine grains. As time goes by, some small grains disappear, as highlighted by yellow circles in Fig. 2. In the case of 0.50*T*_*m*_, three nuclei are nucleated at approximately 200 ps and grow into spherical grains. Other nuclei are then formed from the remaining melt and later-nucleated grains fill the spaces between previously nucleated grains. The obtained microstructure consists of larger grains than those at 0.58*T*_*m*_. It is expected from the snapshots that there is a critical temperature near 0.58*T*_*m*_ at which the incubation time of nucleation becomes minimum. Similarly, the nucleation rate should become maximum and therefore the grain size should become minimum around the critical temperature.

### Nucleation rate

**Figure 3:**
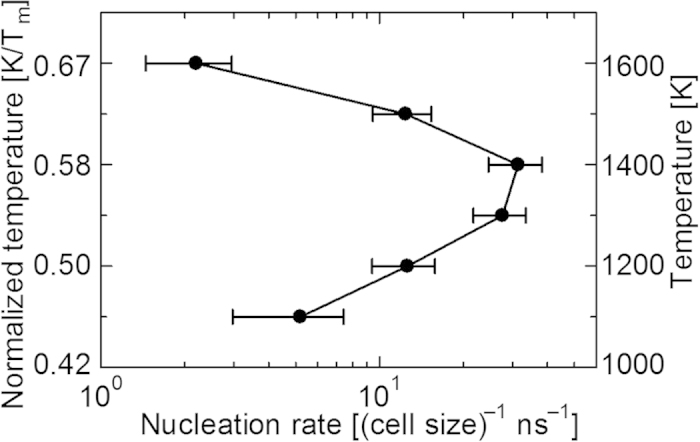
Nucleation rate as a function of temperature. The average number obtained from five replicate calculations for each temperature is plotted with error bars showing the standard deviation.

Nucleation rate per 1 ns, i.e., the number of nucleations in the calculation cell during 1000 ps (1 ns) calculation, is shown as a function of temperature in Fig. 3. The average number obtained from the five replicate calculations for each temperature is plotted with error bars showing the standard deviation. The curve connecting the plots has a characteristic shape with a nose at the critical temperature. According to classical nucleation theory for homogeneous nucleation, the rate of formation of homogeneous nuclei *I* is expressed as^2^


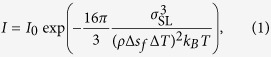


where *T* is the temperature, *σ*_SL_ is the solid-liquid interfacial energy, *ρ* is the density, Δ*S*_*f*_ is the entropy of fusion, Δ*T* is the undercooling temperature (Δ*T* = *T*_m_ − *T*), and *k*_*B*_ is the Boltzmann constant. The prefactor *I*_0_ is the product of the atomic vibration frequency, the probability of capturing an atom at the surface, and the number density of the liquid, none of which are strongly temperature-dependent. Therefore, the nucleation rate strongly depends on the temperature through the two competing term in the denominator of the exponential in equation (1). The nucleation rate increases rapidly with increasing undercooling temperature Δ*T* (i.e., decreasing temperature), which corresponds to a decrease in the nucleation energy barrier. On the other hand, the mobility of atoms decreases with decreasing temperature since the mobility of atoms is assumed to be a thermally activated process with the Maxwell-Boltzmann distribution. These competing effects result in the nose shape when the nucleation rate is plotted against temperature.

Since the nucleation rate as a function of temperature obtained from the MD simulation has a characteristic shape with a nose at the critical temperature, it is expected that thermally activated homogeneous nucleation will occur spontaneously in the MD simulation. The nucleation rate takes a maximum value of 31.5 per 1 ns in the simulation cell at 0.58 *T*_*m*_, which is equivalent to 2.56 × 10^33^ (m^−3^ s^−1^). Since the typical nucleation rate for the homogeneous nucleation of a pure metal near the critical temperature has been estimated to be on the order of 10^30^ to 10^40^ (m^−3^ s^−1^)^2^, the value estimated from the MD simulation is reasonable. In general, it is not straightforward to directly estimate the nucleation energy barrier by the atomic simulation. Regarding this matter, Auer and Frenkel^27^ have discussed the discrepancy in the nucleation rate from those obtained using classical nucleation theory and the hard-sphere model. One of the reasons for the difficulty in estimating the nucleation rate by atomic simulation is the variety of structures in the nuclei. Gebauer *et al.*^28^ examined a prenucleation cluster, which can be amorphous or have different crystal structures from the bulk-phase structure. In this study, only the bcc structure is considered for the nucleus for simplicity, which is the same as the structure in the bulk phase. We note that it is not straightforward to precisely define the structure of a nucleus at a high temperature, which is therefore beyond the scope of this study.

### Incubation time of nucleation

**Figure 4:**
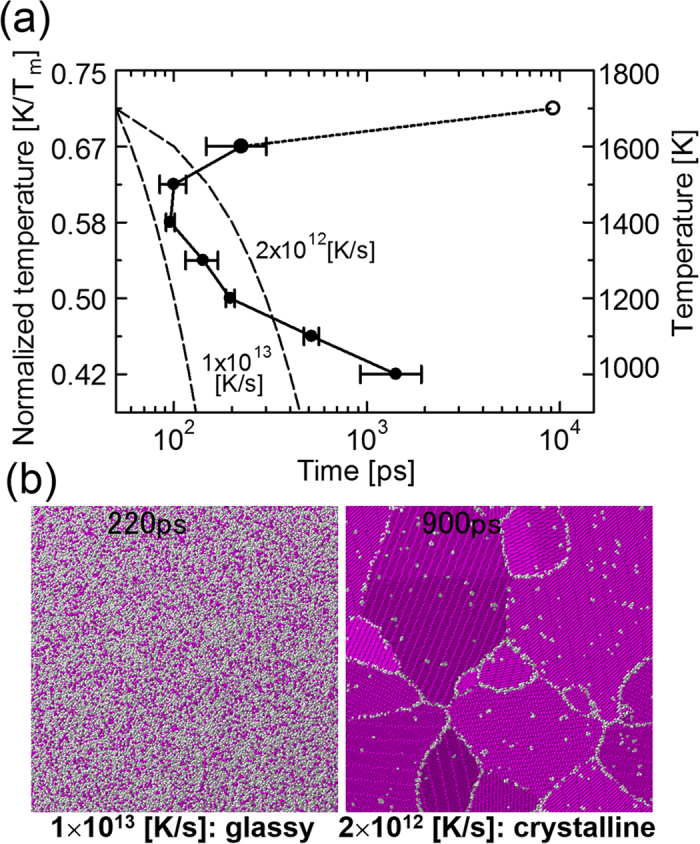
(**a**) Time-temperature-transformation (TTT) diagram. The average number obtained from five replicate calculations for each temperature is plotted (filled circles) with error bars showing the standard deviation except for the case of 0.71*T*_*m*_. For 0.71*T*_*m*_, the actual measurement value from one calculation is plotted (open circle). (**b**) Snapshots of atomic configuration after the continuous cooling transformation (CCT) along dashed lines in (**a**), which represent cooling rates of 1 × 10^13^ and 2 × 10^12^ K/s, respectively.

Next, the incubation time of the first nucleation is shown as a function of temperature in Fig. 4(a). Similarly to the nucleation rate, the average number obtained from the five replicate calculations for each temperature is plotted with error bars showing the standard deviation except for the case of 0.71*T*_*m*_. At 0.71*T*_*m*_, no nucleation occurs in all five replicate calculations over 1000 ps. Therefore, further calculations are performed up to 10000 ps for all five replicate cells. Nucleation occurs at 9250 ps in one of the five replicate calculations, which is plotted in the diagram with an open circle. The curve connecting the plots has a characteristic shape of a time-temperature-transformation (TTT) curve (called the C-curve) with a nose at the critical temperature, as in the case of the nucleation rate. It is known that there is a complementary relationship between the nucleation rate and the incubation time of nucleation^2^, which have a relationship with the mirror symmetry. That is, the incubation time of nucleation *t*_*n*_ is defined by the inverse of the nucleation rate *I* in Equation (1) as^2^


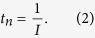


Therefore, the incubation time as a function of temperature also has a nose owing to the two competing factors described above. The above discussion applies to our results in Figs 3 and 4(a), which generally have mirror symmetry, although the critical temperature does not match perfectly.

The TTT diagram is useful for predicting the final structure obtained by continuous cooling of the melt at a constant cooling rate. The dashed lines in Fig. 4(a) show continuous cooling transformation (CCT) lines for cooling rates of 1 × 10^13^ and 2 × 10^12^ K/s across 0.71*T*_*m*_ at 50 ps. The CCT line for 1 × 10^13^ K/s avoids the nose of the TTT curve, and therefore continuous cooling along this line should result in the formation of a glassy structure since no nucleation occurs to the left of the TTT curve. On the other hand, the CCT line for 2 × 10^12^ K/s intersects the TTT curve, which means that a bcc crystal should be formed when the melt is cooled along this line. To confirm this, a further calculation is performed, in which continuous cooling along these CCT lines is assumed. Figure 4(b) shows snapshots of the final structures obtained by continuous cooling along the dashed lines in Fig. 4(a). A glassy structure is formed by continuous cooling with a rate of 1 × 10^13^ K/s, whereas bcc crystalline grains are formed with a cooling rate of 2 × 10^12^ K/s. This result agrees with the prediction from the TTT diagram. Moreover, it is consistent with a previous MD simulation of the solidification of molybdenum nanoparticles^29^, in which the threshold cooling rate for the forming of glassy or crystalline nanoparticles lies between 10^12^ and 10^13^ K/s. On the other hand, this cooling rate is much higher than typical values obtained from experiments^2^. We emphasize here that the cooling rate on the order of 10^12^ K/s is for the case of ideal homogeneous nucleation from the melt of a monatomic metal, whereas most metallic glasses have an alloy composition. Interestingly, in recent experiments, monatomic metallic glasses of Ta and V have been formed through ultrafast liquid quenching with a cooling rate of 10^14^ K/s^30^. Therefore, it is reasonable that the critical nucleation rate for a monatomic metallic glass is on the order of 10^13^ K/s. Moreover, the effect of impurities and other external inducers is not negligible in the most actual experiments, which causes heterogeneous nucleation^2^.

### Microstructure evolution

Snapshots of representative atomic configurations and the grain size distribution after 1000 ps calculation for each temperature are shown in Supplementary Information (Figs S1 and S2). In general, the average grain size becomes minimum around the critical temperature. However, it is not easy to find a clear trend in the grain size distribution from the results, although it is generally known that the grain size has a log-normal size distribution^4^. It is considered that the microstructure obtained after 1000 ps calculation is still undergoing grain coarsening. Therefore, the subsequent microstructure evolution is closely investigated by performing a further calculation up to 10000 ps for the finest grain structure in Fig. 2 (at 0.58*T*_*m*_).

**Figure 5:**
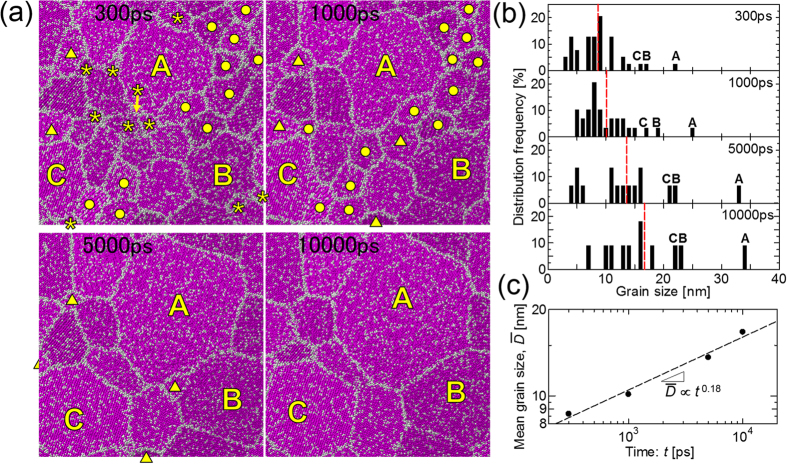
(**a**) Snapshots of the atomic configuration during the microstructure evolution at 0.58*T*_*m*_. Grains labelled by asterisks (*), filled circles (•), and filled triangles (▲) represent those that disappear before 1000, 5000, and 10000 ps, respectively. The largest, second-largest, and third-largest grains are labeled A, B, and C, respectively. (**b**) Grain size distribution directly measured from the snapshots. Red dashed lines represent the arithmetic mean of the grain size. The grain size distribution is normalized by the total number of grain in each step. (**c**) Double logarithmic plot of the mean grain size as a function of time.

Figure 5(a) shows snapshots of the atomic configuration during grain coarsening in the 10000 ps calculation at 0.58*T*_*m*_. The grain size distributions corresponding to the snapshots are shown in Fig. 5(b). It is confirmed from both the snapshots and the grain size distributions that most of the small grains of size less than 10 nm (labeled asterisks, squares, and triangles) that appear at 300 ps shrink and disappear before 10000 ps has elapsed. On the other hand, large grains (such as those labeled A, B, and C) become larger, which can be regarded as grain coarsening. The small grains that disappear at an early stage (labeled by asterisks) are disproportionately distributed in the area where solidification occurs late in the nucleation process as shown in Fig. 2, in contrast to the random distribution. Therefore, grains that nucleate earlier are more likely to survive during the evolution of the microstructure.

As shown in Fig. 5(b), the mean grain size increases during grain coarsening. It is expected from the theory of curvature-driven growth^31^ that the mean grain size follows a power law of the form 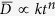, where 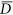 is the mean grain size, *k* is the constant of proportionality, *t* is the time, and *n* is the grain growth exponent. According to the theory of curvature-driven growth, the grain growth exponent is 0.5. Figure 5(c) shows a double logarithmic plot of the mean grain size as a function of time. From the slope of the fitted line, the grain growth exponent is estimated to be approximately 0.18, which is smaller than the theoretical value^31^. However, smaller values (0.375^4^, 0.25^32^) for the grain growth exponent have been obtained by MC^4^,^32^ and phase-field^33^ simulations. The discrepancy in the grain growth exponent has been investigated from various aspects. For example, the anisotropy in the grain boundary energy^32^,^33^ and the mobility^33^ is considered to decrease the grain growth exponent. Moreover, the number of grains should affect the grain growth exponent^5^. In addition, the discrepancy in our case may also be due to the effect of the quasi-two-dimensional system. That is, the mean grain size reaches approximately four times the thickness of the calculation system in our calculation, which have affected the result. Physical factors affecting the grain growth exponent will be investigated in future.

**Figure 6:**
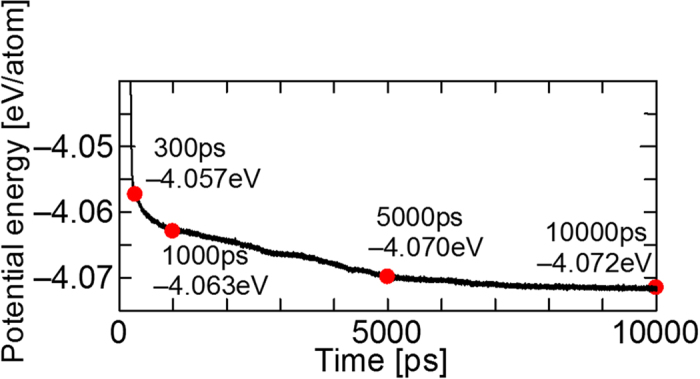
Time variation of the potential energy per atom in the calculation system.

Finally, the potential energy of the calculation system as a function of time is shown in Fig. 6. The potential energy decreases monotonically during the grain coarsening, which is considered to be due to the decrease of the area of the grain boundary, since the existence of a grain boundary causes an excess energy (i.e., grain boundary energy) of 0.5 to 2.0 Jm^−2^ depending on the orientation^26^. Therefore, it is concluded that the decrease in the grain boundary energy is the main driving force of grain coarsening at this stage.

## Conclusion

By the statistical sampling of a million-atom MD simulation, nucleation and the subsequent grain growth in an undercooled iron melt are closely investigated. The nucleation rate and the incubation time of nucleation as functions of temperature have characteristic shapes with a nose at the critical temperature, which indicates the thermally activated homogeneous nucleation. Further calculations along the CCT lines in the TTT diagram yielded glassy and crystalline structures depending on the cooling rate, in agreement with the prediction based on the TTT diagram. Moreover, the subsequent microstructure evolution was investigated by further calculations over ten nanoseconds, from which the grain growth exponent was directly estimated.

Given that most MD studies still achieve nucleation forcibly using an inducing factor for the sake of computational efficiency, it is significant that the spontaneous homogeneous nucleation was achieved in this study without the use of inducing factor owing to the acceleration of the MD simulation, which was performed on a GPU supercomputer. Although a single GPU computation can perform a million-atom MD simulation, multi-GPU computation will increase the possibility of further large-scale simulations. Multi-GPU computation has already been successfully used for a very large scale phase-field simulation of the growth of dendrite assemblages^34^. This technique will be applied in very large scale MD simulation in the near future.

## Methods

### Molecular dynamics simulation

The classical MD method is employed to investigate the nucleation and microstructure evolution from the undercooled melt. The simulation methodology basically follows previous studies^19^,^24^,^25^. The Finnis–Sinclair (FS) potential^35^ is employed for the interatomic potential between iron atoms, which is one of the most established potentials for body-centered-cubic (bcc) metals. It has been confirmed in previous studies that the FS potential can accurately reproduce the thermodynamic and kinetic properties of the solid–liquid interface of bcc metals at high temperatures^16^,^36^. The total energy *E* of the FS potential is expressed as


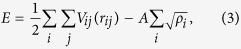



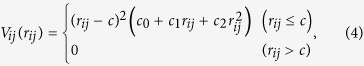



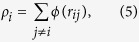



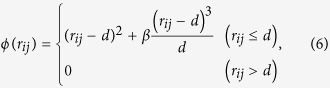


where *V* is the repulsive term, *r*_*ij*_ is the bond length between atoms *i* and *j*, *ρ* is the total electronic charge density at the site of atom *i*, which is constructed by the rigid superposition of atomic charge densities *ϕ*, *A* is the binding energy, *c*_0_, *c*_1_, and *c*_2_ are free parameters used for fitting experimental data, *c* and *d* are cutoff parameters assumed to lie between the second-nearest- and third-nearest-neighbor atoms, and *β* is a parameter used to introduce the maximum value of *ϕ* within the first-nearest-neighbor distance. The parameters for bcc iron from the original FS paper are employed^35^ (Table 1). A leapfrog method is used to integrate the classical equation of motion with a time step of 5.0 fs. A Berendsen thermostat^37^ is applied to control the temperature in each step, and the Andersen method^38^ is applied to independently control the pressure in each direction.

### Calculation procedure

The initial configuration of the calculation system (Fig. 1) is prepared by heating a bcc crystal of iron of size 53.4 × 4.3 × 53.4 nm (186 × 15 × 186 unit cell, 1,037,880 atoms) at 3500 K with the NVT constant ensemble. We note that the calculation system is highly anisotropic in the plane, so is therefore regarded as a quasi-two-dimensional system. The periodic boundary condition is employed for all boundaries. The prepared initial configuration is annealed isothermally in the main calculation for 1000 ps under zero pressure (NPT constant ensemble) at various temperatures between 900 and 1800 K with 100 K intervals. To measure the incubation time of nucleation, further calculations up to 3000 and 10000 ps are performed at 1000 and 1700 K, respectively. For each temperature, five replicate calculations are performed to gather statistics. The main calculations are carried out on the GPU supercomputer, TSUBAME2.5 at Tokyo Institute of Technology with the original code developed with a CUDA 3.0^24^,^25^.

### Configuration analysis

The atomic configurations obtained from the main calculation are closely analyzed as follows to identify the solid and liquid structures. The metal atoms with the bcc configuration are determined by considering the coordination numbers of the first- and second-nearest-neighbor atoms using two cutoff lengths^21^. That is, atoms satisfying the following two conditions are identified as having the bcc configuration: (i) 8 neighbor atoms within a cutoff length of 2.75 Å, which is between the first- and second-nearest-neighbor atoms of the bcc crystal and (ii) 14 neighbor atoms within another cutoff length of 3.4 Å, which is between the second- and third-nearest-neighbor atoms of the bcc crystal. The cutoff lengths were chosen on the basis of our previous study^21^. The atoms defined as being in the bcc configuration are colored in red and the other atoms are colored in white in all figures. Although the determination of the bcc configuration at a high temperature includes an inherent error due to thermal vibration, it is sufficiently accurate to distinguish the bcc crystalline region from the melt and the grain boundaries as shown in the snapshots.

### Image analysis to obtain grain size distribution

The grain sizes in the snapshots are measured by image analysis using ImageJ^39^, which is widely known software for image analysis. Here, the area of a grain *A* is estimated by manually tracing the corresponding grain boundary with a polygonal line. The estimated area is then converted into the effective diameter *D*_eff_ under the assumption that the grain is spherical using


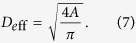


In this study, the effective diameter is adopted as the grain size.

## Additional Information

**How to cite this article**: Shibuta, Y. *et al.* Homogeneous nucleation and microstructure evolution in million-atom molecular dynamics simulation. *Sci. Rep.* **5**, 13534; doi: 10.1038/srep13534 (2015).

## Supplementary Material

Supplementary video 1

Supplementary video 2

Supplementary video 3

Supplementary Information

## Figures and Tables

**Table 1:** Potential parameters employed in the simulation^35^.

*d*[Å]	*A*[eV]	*β*	*c*[Å]	*c*_0_	*c*_1_	*c*_2_
3.569745	1.828905	1.8	3.40	1.2371147	−0.3592185	−0.0385607
