# Supplementary material for: Homogeneous nucleation and microstructure evolution in million-atom molecular dynamics simulation
Source: Sci Rep. 2015 Aug 27;5:13534. doi: 10.1038/srep13534 (PMC4550917; doi:10.1038/srep13534)
Supplement: Supplementary Information [file srep13534-s4.pdf]

Supplementary information for

Homogeneous nucleation and microstructure evolution in million-atom molecular dynamics simulation

Yasushi Shibuta[1]*, Kanae Oguchi[1], Tomohiro Takaki[2] and Munekazu Ohno[3]

[1]*Department of Materials Engineering, The University of Tokyo*

7-3-1 Hongo, Bunkyo-ku, Tokyo 113-8656, Japan

[2]*Mechanical and System Engineering, Kyoto Institute of Technology*

Matsugasaki, Sakyo-ku, Kyoto 606-8585, Japan

[3]*Division of Materials Science and Engineering, Faculty of Engineering, Hokkaido University*

Kita 13 Nishi 8, Kita-ku, Sapporo, Hokkaido 060-8628, Japan

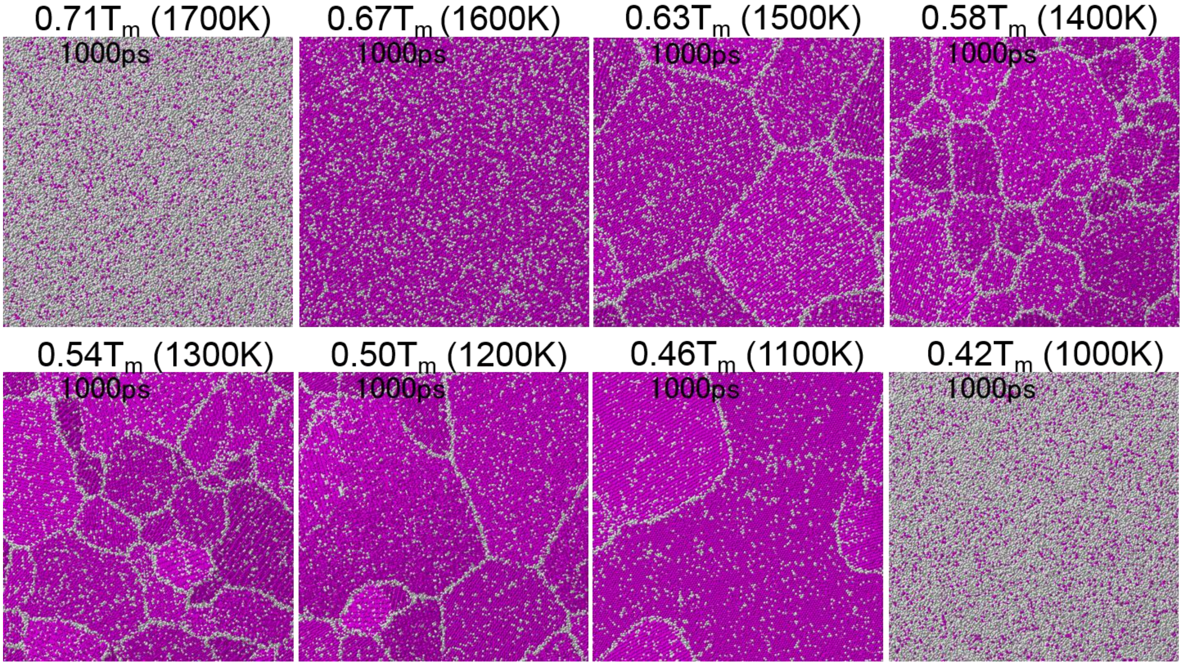

Figure S1. (a) Snapshots of the representative atomic configuration after 1000 ps calculation for each temperature.

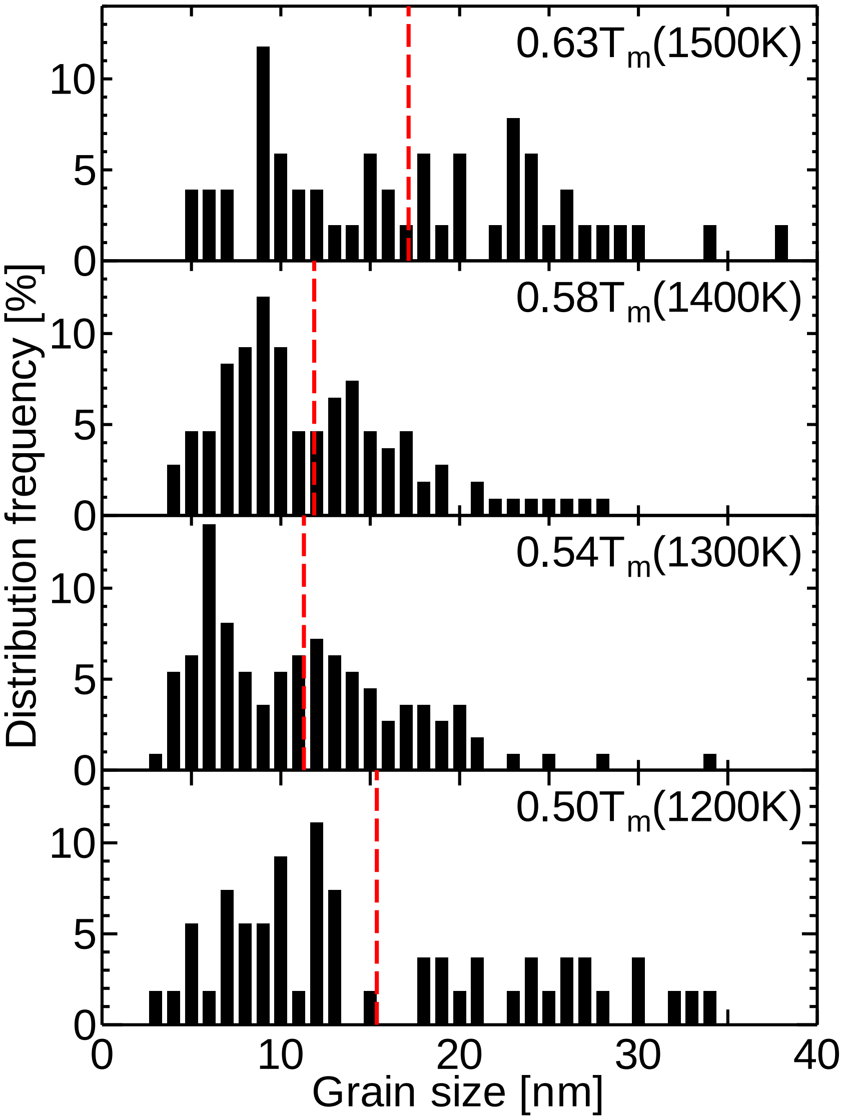

Figure S2. Grain size distribution directly measured from the snapshots at 1000 ps from five replicate calculations for each temperature. The grain size distribution is normalized by total grain number for each temperature. Red dashed lines represent the arithmetic average of the grain size.
